# Towards more effective robotic gait training for stroke rehabilitation: a review

**DOI:** 10.1186/1743-0003-9-65

**Published:** 2012-09-07

**Authors:** Andrew Pennycott, Dario Wyss, Heike Vallery, Verena Klamroth-Marganska, Robert Riener

**Affiliations:** 1 Sensory-Motor Systems Lab, ETH Zürich, Zürich, Switzerland; 2 University Hospital Balgrist, University of Zürich, Zürich, Switzerland

## Abstract

**Background:**

Stroke is the most common cause of disability in the developed world and can severely degrade walking function. Robot-driven gait therapy can provide assistance to patients during training and offers a number of advantages over other forms of therapy. These potential benefits do not, however, seem to have been fully realised as of yet in clinical practice.

**Objectives:**

This review determines ways in which robot-driven gait technology could be improved in order to achieve better outcomes in gait rehabilitation.

**Methods:**

The literature on gait impairments caused by stroke is reviewed, followed by research detailing the different pathways to recovery. The outcomes of clinical trials investigating robot-driven gait therapy are then examined. Finally, an analysis of the literature focused on the technical features of the robot-based devices is presented. This review thus combines both clinical and technical aspects in order to determine the routes by which robot-driven gait therapy could be further developed.

**Conclusions:**

Active subject participation in robot-driven gait therapy is vital to many of the potential recovery pathways and is therefore an important feature of gait training. Higher levels of subject participation and challenge could be promoted through designs with a high emphasis on robotic transparency and sufficient degrees of freedom to allow other aspects of gait such as balance to be incorporated.

## Introduction

Stroke is the third most common cause of death and the biggest contributor to adult disability in developed countries [1]. For instance, around half a million cases of stroke occur per year in the United States [2]. Gait impairment is a large contributor to long-term disability and ambulatory function in daily living [3]. Many patients, however, lose the ability to walk independently, and furthermore, a large proportion do not regain their normal walking speeds following a stroke [4,5].

Treatment for stroke is very costly and accounts for a large percentage of heath care budgets, for example, of the National Health Service in the UK [6]. The approach to stroke physiotherapy is diverse, as are the theoretical bases assumed by the physiotherapists who provide the therapy [3,7-10]. Traditional methodology includes neuro-developmental training (NDT) [11], the motor re-learning programme [12], proprioceptive neuromuscular facilitation [13], and the Rood approach [14]. The effects of the different kinds of training on gait have been shown to be modest, irrespective of the exact type of training [15].

NDT is particularly prevalent [6,7,9,16], with the best well-known stream being the Bobath concept. This therapy attempts a ‘holistic’ approach where emotional, social and functional problems are targeted in addition to the main sensory-motor deficits [11,17]. The general aim is to suppress abnormal movement synergies and move towards normal motor patterns [17]. Despite the acceptance of neuro-developmental training and other conventional rehabilitation techniques, evidence demonstrating their efficacy is lacking [8,10,18-23].

Better outcomes in gait rehabilitation have been elicited from the more direct approach of body weight supported treadmill training [20,24-26], where the patient walks on a treadmill with his or her body weight partially supported, and one or more therapists support the patient and guide their limbs where required. This type of therapy has the advantages of being task specific and repetitive but is often very physically intensive. As a result, the training duration can be limited by the fitness of the therapists themselves.

As a solution to this limitation, robot-driven therapy was proposed as an addition to physiotherapy programs for the neurologically impaired [27]. Robot-driven gait therapy, where the patient is aided by robotic actuators rather than a therapist, is becoming an increasingly prominent feature of rehabilitation worldwide. As well as alleviating the physical load on therapists, a robot can accurately and objectively measure a patient’s output, for example, in terms of joint kinematics and kinetics [28,29].

A number of therapeutic benefits of robot-assisted gait training for stroke patients have been reported. For instance, improvements in walking independence and mobility in the community [20,30], functional walking ability [31,32], muscle activation patterns [31,33], gait speed [33,34], muscle tone [33] and joint range of motions [34] have been shown. Indeed, the robot-based training has been, at least in some cases, demonstrated to be more effective than the more conventional (though more popular) forms of physiotherapy [35,36].

Nevertheless, robot-based therapy has not demonstrated a clear improvement over the manual, therapist-assisted training. Some research has yielded results in favour of the latter [37,38], other results in favour of robot-aided training [32,33] and other studies finding no significant differences in functional gait improvements between the two types of therapy [39-41]. One argument in favour of the robotic approach is that it is roughly as effective as the manual treadmill therapy guided by therapists while requiring much less physical input during its operation [32].

The lack of clear superiority of robot-assisted training has led to some degree of disappointment and even scepticism amongst clinicians. It would seem that despite the purported benefits of the robotic devices, the promise of the training concept and technology has not yet been fulfilled. This review investigates ways in which better designs and more appropriate use of the robotic devices could be made. Firstly, the major factors contributing to the deterioration of gait in stroke patients are described, followed by details of some potential recovery mechanisms by which these impairments could be alleviated, thereby improving walking function. The existing robotic technology for gait rehabilitation after stroke is summarised, followed by an overview of clinical trials investigating robot-driven gait therapy. Finally, elements of the robotic hardware and training needed to maximise the therapeutic impact of the technology are discussed.

## Adverse effects of stroke on gait

Levels of ambulatory activity in stroke patients are low [42]. The adverse effects of stroke on gait function arise from several types of impairment, which is defined as a loss of or deviation from the normal state and function of a body part or organ [43], or alternatively, as a loss or abnormality of physiological or anatomical structure [44]. Disability, on the other hand, refers to the resulting decreased ability to perform a task or function. A stroke thus imparts numerous physical, psychological and cognitive impairments which combine to produce disability [15,29].

Motor, cognitive and sensory deficits are common impacts of stroke [2,6]. A large impact on force and torque outputs has been found for stroke patients, not just for the paretic leg but also for the so-called unaffected leg [45-51]. Lower limb muscle strength has been shown to have moderate to strong relationships with functional walking scores [47,52-59] and also with gait speed [57,60].

Several factors contribute to the overall reduction in strength. Weakness can arise from both activation failure and a reduction in the force-generating capacity of the motor units themselves [49,61]. One important factor in force production diminishment is the reduced number of recruited motor units in the paretic leg [50]. Changes in timing of the muscle groups can also play a role [62], as do reduced conduction velocities of the motor units (of slow-conducting fibres) [50,63], slower torque development and relaxation rates [49-51,54] and poorer fatigue resistance [49,54,61,64].

As well as these adverse effects on the agonists, antagonist muscles can create resistance and thus reduce the net force or torque output. Indeed, stroke patients do show an increased passive resistance to movement. This has been thought to arise from spasticity, an exaggerated tonic stretch reflex due to hyper-excitability of stretch reflexes, resulting in sudden, spasmodic muscular movements [65]. Much effort has thus been made to date in attempting to reduce levels of muscle tone in stroke patients since this was previously thought to be a large contributor to gait disorders [9,66,67]. However, the magnitude of its role in stroke gait disability seems small [47,61,67-69], though some weak correlation of gait indices such as speed and activity scores has been noted [55,70,71], along with data supporting a role of antagonist co-contraction in contributing to motor dysfunction at higher movement speeds [72-75]. In any case, some degree of co-contraction is necessary to maintain joint stability under loading [76], and increased co-contraction of the non-paretic limb may even be a compensatory mechanism used to counteract weakness on the paretic side [76,77].

Effects on force output are not restricted to the nervous system. The sedentary lifestyle that stroke can impose elicits changes in the properties of the motor units such as muscle atrophy, and adverse effects on joints and other tissue [49,78-81]. Increased fat deposition occurs in the paretic leg [80]. Altered structure at the local level, that is of mechanical properties of the musculo-tendinous units and joints, can have an impact on the resistance of the joints to movement [47,66,67,82-84].

The role of abnormal motor templates in stroke has been recognised for many years. Synergies in motor control, possibly encoded at spinal cord [85] or brainstem [86] levels, refer to the use of a basic set of movement combinations in a modular fashion in order to create more complex movement patterns [87-89]. Synergies affect secondary torques, which refer to moments generated about adjacent joints when a person attempts to produce a voluntary torque about a specific joint [90]. Stroke patients often lose independent control of some muscle groups and can only produce movement and torque coupled with other joint moments [61,88,90]. Furthermore, the secondary torques of stroke patients can even be higher than the primary torques from an attempted motor output [90]. Research has indeed shown that some stroke patients seem to be constrained within abnormal motor synergies and have fewer motor patterns [88,91,92]. However, the actual impact of abnormal synergies on gait remains, like spasticity, controversial. The percentage of patients limited by abnormal synergies was shown to be low in one study [93], and in another series of tests, able-bodied subjects and stroke patients exhibited the same synergies in most cases during a standing task [72,74]. It does seem to be the case, however, that kinetic variability amongst stroke patients is much higher than in able-bodied people [77,91].

Sense of touch, temperature and pain can be greatly diminished, particularly on one side of the body. Other impacts on the sensory systems include paresthesia (numbness or tingling sensation) [94-96] and central poststroke pain [95,97]. Impaired sensory abilities may, in turn, also contribute to diminished motor performance, mobility and independence [98,99].

Proprioception at the ankle joint is poor and this may have a role in reducing function [56], for instance through hindering foot positioning and loading during stance [100], degrading the ability to correctly use limb synergies, and also restricting the use of feedback for error correction in motor learning [101]. Poor body positioning and reduced feedback due to proprioceptive loss are likely to impair balance. Nevertheless, the actual impact of proprioception on gait function has not been clearly demonstrated, with some data suggesting that its role in determining function may be weak [102].

Levels of cardiovascular fitness amongst stroke patients are poor [42,70,78], whilst a further frequent secondary complication is a decreased level of bone density [103,104]. The direct constraining effect of these factors on walking function indicated, for instance, by timed tests, seems to be low [41,70]. However, these secondary complications may strongly contribute to a lack of mobility in the community, leading to a more sedentary lifestyle and limiting walking practise and therefore the potential for ambulatory improvement. Relieving the impact of secondary complications is, in any case, an important aspect of stroke rehabilitation: cardiovascular disease is a major co-morbidity in stroke patients [105].

## Pathways to recovery

Different mechanisms can help alleviate the various impairments described above, thereby improving function. Recovery of gait (and indeed other functions) in stroke patients typically occurs within six months of the incident, with most gains being made within three months or less [4,106,107]. Indeed, early commencement of training following stroke tends to yield better rehabilitative outcomes [23,108-111]. Nevertheless, there is evidence that functional gains can continue in the chronic stroke phase [112]. In general, recovery of motor function can occur either through neural reorganisation, which involves finding alternative means to activate the same muscles used for a task prior to injury, or using alternative muscles in compensatory strategies [113].

Some recovery of function and reorganisation does not require training and thus is referred to as spontaneous recovery; this is most prominent until four to six weeks post-stroke [114]. It has been argued that despite the efforts of rehabilitation, the majority of gait recovery following stroke is owed to these spontaneous recovery pathways [115]. Shortly after stroke, some improvements may occur due to recovery of penumbral tissue (brain regions surrounding the insult) [116]. Initially, this tissue receives just enough oxygen to survive, with its function only being resumed later. During diaschisis, also known as apoplectic shock, regions distal from the primary insult are affected due to their connection with the destroyed neurons [117]; recovery may occur to some extent when these interconnections have ceased [118].

Much attention is now being given to the innate plasticity of the nervous system. Previously, the dominant concept was a ‘hard-wired’ view of the nervous system which could be barely altered following damage. The term plasticity refers to the potential for change within the nervous system and includes all reorganisational mechanisms [119], including axonal sprouting, unmasking of previously inactive synapses and the formation of new synapses [120-122]. Reorganisation of brain networks has already been observed in humans [123,124], non-human primates [125-128] and rats [129,130]. Importantly, despite the disrupted motor patterns following stroke, motor system reorganisation has been demonstrated in stroke patients [131-133]; indeed, motor activation patterns can be altered following both peripheral neurological injury [129] and central lesions [124]. The cortical reorganisation which occurs following a stroke has some features normally seen in the developing brain during ortogenesis [134,135].

The neural reorganisation typically involves activation of secondary brain regions such as the premotor cortex and supplementary motor area. The greater the damage to the primary cortical area, the more plasticity can be expected in the secondary areas [136]. However, it seems that these areas are less efficient at producing output than their primary counterparts, and the adverse effects of stroke on function can thus only be partially compensated by such shifts [137]. Patients approaching complete recovery tend to have brain activation patterns which are more similar to healthy controls, with a negative correlation between the degree of recovery and activation of secondary brain areas having been noted [138]. Another typical pattern is the activation of contralesional brain areas [139] usually seen in more severe strokes [136]. The role of this mechanism remains controversial [133], with some studies suggesting a degradation in motor skills from excess contralesional brain activity due to inhibitory drive [140].

The innate ability for reorganisation within the nervous system also raises the question of whether there are catalysts which could be harnessed in a rehabilitation setting in order to maximise gains from plasticity. This has led to interest in the topic of motor learning. Research has suggested that repetitive, task-specific training (as opposed to simply using a limb by chance) is most effective for cortical and task learning reorganisation [126,141-143]. Most improvements will be seen with respect to the specific task that is trained, with only limited generalisation to other movements. These facts suggest that acquisition of specific skills via motor learning is a crucial element of neural reorganisation [113].

For motor learning of a new task, training which incorporates active participation where the subject voluntarily produces a movement is thought to be essential for inducing changes in motor performance, cortical activity and excitability [144]. Correct afferent input is another important factor in motor learning [145], with more severe sensory losses being associated with higher levels of motor dysfunction in stroke patients, possibly because the lack of sensory input acts a barrier to motor learning and reorganisation [146]. It has been suggested that sensory feedback is needed for processing by internal models which can predict and adjust motor outcomes [147], implying that correct sensory input is essential for motor adaptation through training [119]. A further ingredient required for motor learning is the inclusion of variation during the training or practice. This can improve retention of a newly-learned skill [148], and also promote generalisation, thus improving performance in other tasks to some degree. Moreover, higher intensities of practise tend to result in better outcomes in motor learning [149].

Rehabilitative training has produced reductions in the severity of a number of impairments of stroke patients. For instance, rehabilitation has helped to increase the strength of the paretic lower limb [150,151]. A pooling of various studies has revealed a significant and homogeneous effect of transcutaneous electrical nerve stimulation on muscle tone [23], and improvements in stroke patients’ cardiovascular fitness have also been achieved through training [25].

However, reductions in impairment do not necessarily lead to improvements in gait function. Therapy targeting a specific impairment such as muscle strength can produce effects limited to that area only and thus can often fail to translate to improvements in function [20,23,152]. In general, greater improvements at the neuromuscular level (for example, in terms of muscle strength) have been reported than advancements made in function [153]. Even if small gains in function are made, these may not be sufficient to improve a patient’s mobility within the household and community.

Nevertheless, some degree of beneficial impact on walking function has been achieved. Cardiovascular, aerobic and endurance training can have positive effects on gait speed and function [23,154-156]. Studies investigating strengthening of the paretic limb have revealed small effects on gait speed and on gait endurance [23,157]. By way of contrast, functional recovery does not seem to be strongly associated with changes in muscle tone [20].

Results concerning the properties of training required to induce functional improvements in walking are still scarce. For instance, the relationship between rehabilitative outcomes and training dosage remains unclear. While some research has shown a small effect of training intensity on rehabilitative outcomes [108,153,158], doubling the training dosage did not result in a significant improvement in outcomes (for instance, timed walking) in one study [159], and a negative correlation between training dosage and degree of recovery has even been reported [160]. The training dosage seems to be important only where the therapy is functionally-focused - it then becomes a significant (but still quite small) predictor of the outcome [161]. These results thus suggest that the type and nature of the training may be more important than simply the quantity alone.

One hitherto-neglected aspect related to functional gains is that stroke patients tend to adapt to the limitations of their limbs’ motor output by using alternative, still-preserved motor functions through the use of compensatory strategies. Perhaps the most familiar example found in stroke is the use of increased hip abduction to compensate for weakness in the sagittal plane to enable foot clearance during gait. Compensatory mechanisms have been shown to be one of the primary routes to functional recovery for primates [162-164]. Furthermore, some results show that improvements typically occur through tuning of these abnormal synergies rather than by replacing them with a ‘normal’ physiological synergy set [165]. Nevertheless, it is usually the main goal of both clinical rehabilitation and indeed much of rehabilitative technology to strive for the regular motor activation patterns observed in healthy subjects.

Given the above evidence, the best results in terms of gains in walking ability would be expected for task-specific training that incorporates actual walking practise. Given the above impairments and their impact on walking, elements such as balance, including weight bearing, trunk position and lateral movement, joint control, and perhaps non-level walking such as on stairs, gradient climbing and different walking surfaces should be included due to their importance in actual overground walking and their potential to increase training variability and challenge.

However, the various robotic devices and the way in which they are employed in clinics may not provide a suitable means for addressing all these aspects within rehabilitation, and hence may not yet be able to optimally promote the various recovery routes detailed above. The following section is a critical review of some of the existing robotic gait technology.

## Existing robotic gait training technology

A number of devices, some of which are available as commercial products on the market, have been applied in stroke rehabilitation. These can be broadly classified as stationary and overground walking systems.

Regarding stationary walking systems, the Lokomat and the AutoAmbulator are commercially-available devices which support patients to perform walking on a treadmill. The Lokomat consists of an exoskeleton in combination with a body weight support system, and controls the joint angles at the knee and hip by means of linear actuators [27]. The AutoAmbulator (HealthSouth, Birmingham, U.S.) employs robotic arms attached laterally to the patient for control of the lower limbs [166]. These devices share similar limitations: movements are constrained to one anatomical plane (sagittal) which prevents meaningful balance training. Furthermore, there is limited patient influence on the walking trajectory. The patient is often guided through the movements to a large extent, rather than producing them volitionally, though control strategies offering more scope for patient influence on the walking trajectory are being developed [167,168].

There are several alterative, non-commercial treadmill-based, stationary robots, such as the Lower Extremity Powered Exoskeleton (LOPES) system [169]. This was designed with a high emphasis on lightweight design and the incorporation of passive mechanical elements. Translational movements of the pelvis are included along with extension-flexion and abduction-adduction, although an active ankle orthosis is not yet incorporated. The Ambulation-Assisting Robotic Tool for Human Rehabilitation (ARTHuR) is an end-effector system where leg movements are controlled via moving coil forcers [170]. The device can be used with the Pelvic Assist Manipulator (PAM) which allows movement of the pelvis in five degrees of freedom [171]. The Active Leg Exoskeleton (ALEX), in common with the ARTHuR, was designed for high backdrivability^a^, although it does not employ the series elastic elements found in the LOPES. It allows both extension-flexion and abduction-adduction movements of the hip, knee flexion-extension and ankle inversion-eversion [172]. Only the sagittal plane hip and knee movements are actively controlled via linear drives, with the other degrees of freedom being passively supported by springs. Four degrees of freedom are included for the trunk. One of the goals of ALEX is to reduce robot interference on gait by counterbalancing gravitational forces on the legs. Such a compensation strategy can, however, greatly distort the dynamics of gait away from those encountered in normal walking [173]. The Actuated Compliant Robotic Orthosis (ALTACRO) [174,175] uses pleated pneumatic artificial muscle [176] to generate linear motion for control. The prototype device currently provides active assistance for the knee joint only, though it is intended that the device will be extended for active hip and ankle support as well. The AnkleBot has been developed by MIT and provides three degrees of freedom at the ankle, with two of these being actuated (dorsi-plantarflexion, and inversion-eversion), and is designed to be lightweight and backdrivable [177,178].

In addition to the treadmill-centred technology, an alternative approach is to use foot plates to guide the feet and thereby reproduce gait trajectories. The Gait Trainer (Reha-Stim, Berlin, Germany) uses a crank and gear system to guide the feet, simulating stance and swing phases [179]. The system can provide a varying degree of support to the patient, as can the Haptic Walker [180], which is designed for more arbitrary movements of the feet to simulate walking on different surfaces. The GaitMaster [181] and G-EO Systems [182] are robotic end effector devices which allow simulation of stair ascent and descent. The LokoHelp (Medburg, Basel, Switzerland) is a lower leg orthosis system driven by a treadmill rather than external drives [183]. The pelvis is unconstrained, though the feet follow a fixed trajectory. These technologies tend to focus on movements in the sagittal plane; furthermore, the absence of an exoskeleton structure does not allow support of the knee joint which may be challenging for some stroke patients, though this omission does provide an opportunity for assistance and input from the therapist.

As an alternative to the static gait training offered by the above-mentioned platforms, some robotic devices are mobile and thus offer overground walking. The KineAssist (Kinea Design, Evanston, U.S.) has a mobile base and provides partial body weight support and assistance for movements of the pelvis and torso, whilst leaving the patient’s legs unobstructed in order allow therapist assistance [184]. The device can be used for both balance and gait training. The WalkTrainer (Swortec SA, Monthey, Switzerland) uses a motor to follow the movements of the patient and has a parallel robotic structure to control the motion of the pelvis in six degrees of freedom [185]. The training of balance during gait is difficult due to the restrictive hip movement in abduction, however. The Hybrid Assistive Leg (HAL) is a wearable system consisting of an exoskeleton driven by electric motors [186]. The device may be less suitable for the more severely impaired patient groups since limited support for the trunk and pelvis is provided by the wearable structure and there is no actuation in the frontal plane.

Therefore, several trends, including designing for transparency and incorporating more flexibility and degrees of freedom, seem to be apparent in the hardware development. The next section summarises key clinical results regarding the efficacy of robotic training in gait rehabilitation for the stroke population.

## Clinical outcomes of robotic gait technology

The following section describes training outcomes in different groups of stroke patients: effects in acute, subacute and chronic groups are outlined. Most investigations have thus far been pilot studies, though some comparative studies have been carried out for the Gait Trainer and the Lokomat.

Positive effects of robotic training interventions have been demonstrated for patients in the acute phase of stroke. A pilot study comparing the effects in acute patients of a combination of robotic and conventional therapy with the same volume of training of only the latter approach found significant improvements in functional ambulation for both training groups, but no significant differences between the two intervention groups [39]. In tests investigating the Gait Trainer, patient groups trained with the more task-specific robot-driven gait or overground walking training modes (but who were also given conventional therapy) showed greater improvements in functional walking than the conventional therapy group [187]. Nevertheless, it is noteworthy that the patients in the Gait Trainer group rated their perceived exertions lower than the overground walking group.

Subacute patients in one study achieved better independent walking scores and also neurological assessment results from Lokomat training in combination with regular physiotherapy as opposed to an equivalent volume of conventional therapy alone [35]. Nevertheless, no significant differences in timed walking tests between the intervention groups were noted. Similarly, a study with subacute patients across German rehabilitation centres found more improvements in independent walking and secondary variables such as speed and endurance in a patient group that received a blended training approach consisting of the Gait Trainer and conventional physiotherapy [30]. Conversely, another study with subacute patients, again comparing Lokomat training with a more conventional therapy approach, found that the latter training method produced greater performance gains in timed walking tests; however, no differences in functional ambulation between the groups were found [37]. The fact that the first two of these studies (reporting a positive outcome for the robotic training) applied a combination of robot-based and conventional therapy as opposed to robot therapy alone may be one cause of the different conclusions of these investigations. In a further study, greater gains in timed walking tests and motor scores were elicited from Lokomat training than conventional therapy [33]. A trial into the effects of the AutoAmbulator in patients admitted with stroke less than 12 months prior to testing found improvements in both the robotic and conventional therapy groups in timed walking tests, but no significant differences between groups [94]. An investigation into using the Gait Trainer for subacute patients found improvements from the robotic training as well as the manual treadmill therapy in terms of functional walking; no significant differences at six months follow-up were apparent between the groups [32].

Regarding the effects of the training in chronic patients, no significant differences in self-selected walking speed between Lokomat and manual treadmill therapy were found in one investigation [41], though some pre- versus post-test differences were observed in the Lokomat group. By way of contrast, another study employing the same number of sessions and training duration (12 sessions, each of 30 minute duration) found greater improvements in stance time and walking speed through therapist-aided as opposed to robot-assisted training [188]. The authors suggest that the lower intensity of the Lokomat walking as compared with manual treadmill training, as previously highlighted [189], is one of the causes of the lower efficacy of the robotic intervention for chronic patients. A small study of chronic patients training in the LOPES found significant gains in walking speed and distance, and also in joint range of motion [34].

In summary, it seems that the potential of the current robotic technology is greatest in the earlier stages of stroke. While acute stroke patients can benefit from the higher degree of support from the robotic training, there is not such a clear trend in favour of one or the other training approach for chronic patients. Furthermore, the best results have often been noted where the robot was applied in conjunction with conventional therapy, which seems to support the view that the robot is an augmentation to rather than a replacement for the therapist [190].

## How could robot-assisted gait therapy be improved?

From the discussion on neuroplasticity and motor learning, it appears that the level of active participation has a strong impact on almost all of the elements of gait recovery following stroke. However, the voluntary contribution of patients during much of robot-assisted walking has been quite limited so far. Active participation is strongly influenced by the mechanical properties of the robot, the control system, the patient’s motivation, the therapists’ instructions and various other factors.

Arguably, the biggest determinant of how much active participation is ultimately possible is the transparency of the device. Transparency refers to the degree to which the interaction forces and torques between human and robot are generated - that is, the ability of the robot to ‘get out of the way’ [191]. Thus, a perfectly transparent robot would induce no forces on the human subject, and conversely, a robot with low transparency would always distort the walking dynamics and thus impose false loading patterns. A transparent robot allows the level of robotic guidance to be varied, and patients can be exposed to the actual dynamics of the task to a greater extent, which is likely to aid motor learning [192,193]. At the other end of the spectrum, the robot must still be able to perform the basic function that it was originally designed for, that is, aiding the patient in areas where he or she is most impaired and cannot perform independently.

Control of the robot is a crucial element and heavily influences, for example, the overall safety, the level of patient participation, and robotic transparency. The control strategies employed for gait training robots can be divided into a number of categories including assistive and challenge-based controllers [194]. The former case includes the oft-applied impedance control regime [195], which can ensure correct kinematics, but may impair motor learning due to reduced patient effort. Hence, there has been interest in assist-as-needed paradigms where the robot only interferes in phases which cannot be performed independently by the patient [196,197]. In the ideal case, the robot would exert no interference unless the patient is unable to perform a task or gait phase. On the other hand, challenge-based controllers are those which make a function or movement more difficult than the real task [194], and include increased-resistance strategies and error augmentation [198,199]. The latter is based on the assumed importance of error correction in motor learning, with the hypothesis that error amplification leads to an increased rate of motor skill acquisition [200]. Nevertheless, the actual influence on motor learning outcomes remains unclear [200,201], and furthermore, the long-term changes of using this kind of strategy are still unknown [194].

Although influential, control design and implementation can have only a limited effect on the overall level of transparency. The interaction between the robot and the human imposes additional restrictions on the control of the robot [202-204], thereby limiting the extent to which robot inertia, in particular, may be compensated. As a result, the limit of achievable transparency is determined by the hardware of the robot rather than through control [203]. Mechanical designs leading to a bulky, high inertia machine will yield a low transparency device, regardless of the control strategy used. Other potentially problematic aspects of the mechanical design include gearing and actuators which can introduce friction and backlash, impose control bandwidth restrictions and complicate design for safety [205,206].

With this in mind, attempting to keep the various components - including the actuators, joints and exoskeleton - as lightweight as possible is perhaps the most crucial aspect of ensuring sufficient transparency for effective training. Transparency is now being recognised as an important design element: high levels of transparency have featured in realisations of robot systems for rodents [207] and humans [169], with friction and inertia being key considerations. Detaching the motors from the robot frame has been one approach to decreasing the weight and inertia of the moving elements of the robot [208].

The incorporation of passive mechanical elements into the design can also help alleviate various mechanical problems. For example, series elastic actuators (SEA) can act as low-pass filters to reduce shock [205], and since the force output is proportional to the spring length, force control can be approached via position regulation [205,208]. They can also be useful for energy storage and lower the reflected inertia and output impedance [205,206,209]. Furthermore, their higher compliance can reduce the risk of injury in the event of a collision [206]. These various characteristics make the SEA a useful inclusion in robotic exoskeleton applications [205] and it has already found some application in robot-assisted technology [208].

Another source of the limited effectiveness of some of the available robots is thought to arise from limitations in the robotic degrees of freedom. For instance, the constraints on the pelvis imposed by some devices induce changes in the gait kinematics [37,210,211]. The constraint to one anatomical plane (normally sagittal [27]) can severely limit frontal and transverse plane rotations [211]. Restrictions in terms of the robotic degrees of freedom may also force the patient to perform compensatory movements in the sagittal plane, causing muscle activation patterns to be forcibly altered [189,212].

During normal gait, humans must actively control balance in the frontal plane through manipulation of the position of the centre of mass using foot placement [213-215]. Training of balance during walking is not realistic in many of the current devices due to the aforementioned limited degrees of freedom. For example, weight shifting from one leg to the other and the cyclic lateral movements of the centre of mass - the main component of balance during gait [216-218] - are not permitted, and thus the subject need not actively stabilise himself [211]. Even when sufficient degrees of freedom are provided at the pelvis, the inability to perform sufficient hip abduction in some of the machines [185] can also be a barrier to balance training. Additionally, the body weight support systems normally used during the training can further reduce the challenge of maintaining balance due to the stabilising forces they provide [219]. Studies have examined the impact of incorporating standing balance in training on rehabilitation outcomes for stroke patients [220,221]. This, however, is a fundamentally different task from maintaining balance during gait, and therefore the potential improvements in walking function available through including active balance control during robot-assisted gait are still unclear.

While insufficient degrees of freedom can impede certain aspects such as balance practise, it is important to note that simply increasing the number of degrees of freedom will not necessarily lead to enhanced training outcomes. The weight of the additional actuators and joints required may greatly increase the inertia with respect to the various robotic degrees of freedom and may thus conflict with other design objectives such as transparency.

While is clear that there are a number of factors which could potentially have a large impact on training outcomes, there exists only limited evidence to verify the impact of these aspects on actual recovery. For instance, while assist-as-needed controllers have enabled patients to be more active and have decreased the interaction forces between the human and robot [167], research showing that better functional outcomes will follow from their use in training programs is still needed. Data from spinal cord injured rats do imply a useful role of assist-as-needed paradigms in recovery [222], but much more supporting evidence is necessary. On the same theme, data verifying the ability of motor learning after neurological injury to improve the long-term gait function of stroke patients would provide invaluable guidance for the future design of the devices, and propel the motor learning concept from theory into actual clinical application.

## Conclusions

A stroke can cause a number of impairments within various systems, giving a complex underlying structure to the resulting disability. The mechanisms responsible for recovery and reversal of these impairments are still only partially understood. Whilst stroke rehabilitation can have positive effects, its impact on gait recovery remains modest, regardless of the particular methodology applied during rehabilitation.

One of the most important features of any training is that the patients should be as active as possible. This can affect not only the possible gains in motor learning but could also potentially help reduce the severity of the secondary complications of stroke patients such as cardiovascular disease and osteoporosis. A robot designed for a high level of transparency would enhance active patient participation and achieve low robotic interference for the more able patients, whilst retaining the option of more support when it is really needed. A compact, low weight design incorporating passive elements and sufficient degrees of freedom to allow different aspects of gait to be trained will help achieve a high level of voluntary effort on the part of the patient, enhance training variation and thus promote more effective motor learning.

Within this article, a number of concepts have been outlined which have the potential to improve gait rehabilitation outcomes, with the foremost of these arguably being motor learning and neuroplasticity. However, there is a paucity of clinical evidence showing that these concepts can really lead to improved walking function in the long term. Robots have recently been, and continue to be developed which incorporate more degrees of freedom, allow more aspects of gait such as balance to be trained, and which have greater transparency. These will provide an important opportunity in the near future to intensively investigate the clinical impacts of these various concepts. The results of such studies will then be pivotal in guiding future robot design and application, allowing subsequent technological development to take place under the guidance of better defined clinical requirements.

## Endnotes

^a^Backdrivability refers to the ease with which the external world can alter the state of the robot [223].

## Competing interest

The authors declare no competing interests.

## Authors’ Contributions

AP reviewed the technical and clinical literature and drafted the manuscript. DW, HV and RR provided input concerning the technical aspects while VKM gave input on the clinical aspects. All authors have read and approved the final manuscript.
